# Establishment and genomic validation of novel patient-derived xenograft models for drug discovery in gastrointestinal stromal tumor

**DOI:** 10.1186/s12885-026-16659-y

**Published:** 2026-08-10

**Authors:** Marya Kozinova, Ashot Nazaretian, Martin G. Belinsky, Yan Zhou, Shalina Joshi, Karthik Devarajan, Kathy Q. Cai, Delia Zumpano, Shuai Ye, Yinfei Tan, Sanjay S. Reddy, Jeffrey M. Farma, Margaret von Mehren, Lori Rink

**Affiliations:** 1 Cancer Signaling and Microenvironment Program, Fox Chase Cancer Center, 333 Cottman Avenue, Philadelphia, PA 19111-2497 USA; 2 Department of Biostatistics and Bioinformatics, Fox Chase Cancer Center, Philadelphia, PA USA; 3 Histopathology Facility, Fox Chase Cancer Center, Philadelphia, PA USA; 4 Molecular Diagnostics Lab, Department of Pathology, Fox Chase Cancer Center, Philadelphia, PA USA; 5 Department of Surgical Oncology, Fox Chase Cancer Center, Philadelphia, PA USA; 6 Department of Hematology and Oncology, Fox Chase Cancer Center, Philadelphia, PA USA; 7 Biomedical Sciences Graduate Program, Lewis Katz School of Medicine at Temple University, Philadelphia, PA USA

**Keywords:** GIST, PDX, TKI

## Abstract

Most gastrointestinal stromal tumor (GIST) patients develop clinical resistance to approved tyrosine kinase inhibitors (TKI). The development of effective treatment strategies for refractory GIST requires identification of novel targets for investigational agents and well-characterized preclinical models, like patient-derived xenograft (PDX) models, to evaluate them. While GIST PDX models have been shown to mirror patient tumors in cell morphology and driver mutation status, studies across tumor types indicate that PDX models may undergo limited genomic changes during engraftment and passaging. We performed a comprehensive genomic analysis of six *KIT*-mutant GIST PDX models derived from TKI-refractory patients. Pairwise comparison of copy number variation (CNV) profiles indicated strong correlation between PDX and matched primary tumors. Four models exhibited changes in the number of CNV events relative to the primary tumors, suggesting clonal selection and tumor heterogeneity during engraftment and passaging. Analysis of cancer-associated gene aberrations showed stability of primary *KIT* driver mutations, with minor fluctuations in the allelic frequencies of acquired mutations, suggesting initial heterogeneity of GIST tumors and potential enrichment of sub-clonal populations through passaging. To functionally assess model fidelity in vivo, three PDX models were treated with the approved second-line TKI sunitinib. The models exhibited differential responses that could not be fully explained by driver mutation status alone, highlighting the need for further investigation in larger cohorts.

These findings highlight the importance of comprehensive genomic characterization of PDX models prior to their use in preclinical studies, supporting more accurate interpretation of experimental results and preserving their value as research tools.

**Supplementary Information:**

The online version contains supplementary material available at https://doi.org/10.1186/s12885-026-16659-y.

## Introduction

Gastrointestinal stromal tumors (GIST) are the most common connective tissue tumors in the gastrointestinal tract, with an annual incidence of 11 to 19.6 per million [1, 2]. The majority of GIST cases are caused by gain-of-function mutations in the tyrosine kinase receptors *KIT* (70–80%) or *PDGFRA* (5–10%) [3, 4], that in turn activate downstream signaling pathways which lead to cell growth and proliferation. The tyrosine kinase inhibitors (TKI) imatinib mesylate (IM), sunitinib, regorafenib, and ripretinib are approved therapies for advanced or unresectable *KIT*-mutant GIST. IM has transformed GIST therapy, with initial tumor response in > 80% of patients. Despite this, most patients progress within two years. Responses to post-IM lines of TKI therapy are brief (3–9 months). Therapy-resistant GIST may manifest acquired *KIT* or *PDGFRA* mutations, mutations in other kinases, copy number alterations, and/or changes in kinase expression. The development of effective treatment strategies for refractory GIST requires the identification of additional therapeutic targets for approved or investigational agents, and well-characterized preclinical models to test these agents.

One of the major limitations facing the field is the lack of experimental models that recapitulate the features of human GIST including the diversity of molecular sub-types of GIST, tumor heterogeneity, and tumor microenvironment. Investigational models for understanding and treating human cancer have evolved from two- and three-dimensional cell lines and cell line-derived xenografts to patient-derived xenograft (PDX) models. PDXs, from fresh patient tumor tissue directly subcutaneously implanted into immunosuppressed mice followed by tumor growth and subsequent repassaging in mice, have the potential to better preserve the histological features, molecular complexity and intratumoral heterogeneity of the parent tumor [5, 6]. PDX models have been shown to mirror the patient tumor in terms of cell morphology, tumor subtype, driver mutation status, and general patterns of genomic aberrations and gene expression [7–13].

Recent studies underscore the need to rigorously characterize PDX models, as they exhibit both strengths and limitations as preclinical systems [14–16]. While PDXs generally better recapitulate tumor gene expression than cell lines, engraftment induces notable transcriptional changes, particularly in extracellular matrix and immune pathways [13]. Multi-omic analyses show that PDXs largely mirror parental tumors but can acquire new somatic mutations and shifts in clonal composition, reflecting selection of subclonal populations during engraftment and passaging [11, 15]. Similarly, large-scale CNV analyses reveal gains and losses of large genomic regions, with ~ 10–20% divergence from primary tumors that may increase over passages [17–18]. These genomic alterations may influence therapeutic responses and complicate interpretation of preclinical findings.

To date, a large number of GIST PDX animal models have been reported [19–30]. While these PDXs were generally shown to recapitulate the key features of matched patient tumors, such as histological morphology, KIT staining and preservation of the original driver *KIT* mutations, none of these models have been comprehensively profiled at the genome-wide level. In the clinic, GIST manifests a variety of driver mutations along with acquired mutations and other aberrations that develop following multiple lines of therapeutic TKI failure. Recent studies suggest that tumor behavior and therapeutic response may also be influenced by differences in signaling network activity beyond driver mutation status [31]. This diversity creates a demand for establishing and characterizing PDX models that maintain fidelity to the patient tumors and can be utilized for genotype-specific preclinical studies, identification of pathway components that are suitable for therapeutic targeting and the development of personalized patient treatment.

The current study describes molecular-genomics profiling and comparison of patient GISTs to the established and serially passaged PDXs that were derived from these tumors. We successfully established six PDX models from recurrent metastatic refractory GIST and maintained them in vivo over multiple passages in mice. Along with *KIT*/*PDGFRA* genotype determination, histochemical and immunochemical analyses and comparisons of patient to PDX passaged tumors, we performed genome-wide copy number (CN) analysis to define and compare chromosomal aberrations, as well as targeted next-generation sequencing (NGS) profiling with Illumina TruSight Oncology 500 (TSO500) [32], to detect additional mutations in *KIT* and other cancer-related gene changes comparing primary patient tissues to early and late PDX passages. We performed in vivo testing using selected PDX models to evaluate response to the standard therapy sunitinib as a single agent to determine whether these models recapitulate clinical responses.

To our knowledge, this study provides one of the most detailed molecular characterizations of established GIST PDX models compared to the matched patient tumors they derive from, providing insights on tumor stability and possible clonal changes occurring during PDX propagation over time. While PDX models are widely used in translational research, detailed molecular characterization of these models is often limited. Building upon previously reported GIST PDX platforms, our analysis integrates genome-wide copy number profiling with targeted next-generation sequencing to evaluate the preservation of key genomic features across passages, and to highlight the importance of a thorough characterization of PDX models prior to their use in preclinical studies. Thoroughly characterized PDX models have the potential to improve the quality of both preclinical testing of novel TKIs and to provide further insights into resistance mechanisms to these agents. The PDX models described in this study expand upon our previously reported work and are available to the research community upon reasonable request, together with the accompanying genomic data, to support the use of well-characterized models in future GIST research.

## Methods

### Case description and PDX generation

Patient tumor tissues were collected following informed consent with IRB approval (22-9911) by the Fox Chase Cancer Center Institutional Review Board. Fresh surgically resected tumor samples were collected from patients with advanced GIST, minced on ice, mixed with Matrigel and injected subcutaneously into NSG mice, purchased from The Jackson Laboratory. Studies involving animals followed procedures approved by the FCCC Institutional Animal Care and Use Committee (FCCC IACUC protocol #10–16) as previously described [27]. Once established, PDXs were serially transplanted in mice with passage numbers assigned beginning with the initial transplantation into mice (P0).

### GIST PDX and drug administration

PDXs from cases 2, 3, and 6 were established in both male and female mice. The growth of established tumor xenografts was monitored at least twice a week by vernier caliper measurement of the smallest and largest tumor’s diameters and tumor volume was calculated using the formula: tumor volume (mm^3^) = smallest diameter^2^ × largest diameter × (π/6). Mice were randomized into two treatment arms: arm 1, vehicle (5 days/week, oral) and arm 2, sunitinib at 20 mg/kg (5 days/week, oral). Drugs were administered by oral gavage for 21–23 days until the end of the study or until tumors exceeded > 10% of their body weight or demonstrated distress or weight loss > 10% as per the local IACUC guidelines. Euthanasia was performed by CO_2_ inhalation by authorized personnel in accordance with approved protocol. Tumors were harvested and frozen in dry ice. 

### Histology and immunohistochemistry

Hematoxylin & eosin (H&E) and IHC of KIT and DOG1 expression were performed as previously described [33, 34].

### Preparation of tumor genomic DNA for genome-wide copy-number analysis and targeted NGS

Patient tumor specimens and patient-derived xenografts (PDXs) were snap-frozen in liquid nitrogen and stored at -80 °C until use. Grossly viable, tumor-rich regions were selected for DNA extraction, while visibly necrotic or non-tumor tissue was avoided. In the case of PDX samples, care was taken to procure a core segment from the interior of excised tumors avoiding the capsule and adjacent host tissue when possible to minimize host contamination. The isolation, quantification, and characterization of high-quality genomic DNA from flash-frozen tumors have been described [33].

### Copy-number analysis

Copy-number analysis was performed in the Fox Chase Cancer Center Genomics Facility using the CytoScan HD Suite (Thermofisher Scientific, Waltham, MA). The CytoScan HD arrays consist of ~ 2.7 million SNP and non-polymorphic probes. Arrays were processed according to the manufacturer’s instructions. Briefly, total genomic DNA (250 ng) from each test sample is enzyme-digested, adapter-ligated, and amplified with adapter-specific primers under PCR conditions optimized to preferentially amplify fragments in the 150- to 2,000-bp size range. The PCR amplification product is purified via magnetic beads, fragmented, biotin-labeled, and hybridized to a CytoScan™ HD array. The hybridized array is washed with Affymetrix GeneChip Fluidics 450, scanned with GeneChip Scanner 3000 7G, and the intensities of probe hybridization analyzed by the Affymetrix GeneChip Command Console (AGCC).

Data from the CytoScan arrays were analyzed by the Fox Chase Biostatistics and Bioinformatics facility. Probeset level data files generated by the AGCC were analyzed with the Bioconductor rCGH package [35] to produce log2 relative ratios (LRR). Genome-wide LRR were rescaled, a circular binary segmentation algorithm (CBS) [36] was used for segmentation, and data was then normalized and recentered. Cutoffs of 0.55 and − 0.55 were used to define copy number gain and loss. Default size of 10 kB was used to identify discrete CN segments. Segments were manually joined using the output segmentation tables and visual inspection of genome-wide CN plots. We reported the average LRR for each gene, which quantifies the copy number changes in the samples.

### TSO500 next-generation sequencing and analysis

*Illumina TruSight Oncology 500 Assay* (Illumina, cat# 20028213). Briefly, 40 ng genomic DNA was sonicated by the Covaris S2 at Duty factor 10%, 200 cycles per burst for 280 seconds at 7°C. End repairing and A-tailing were performed at 30°C for 30 minutes to convert 5’ and 3’ overhangs into blunt ends and to add A-tail to 3’ ends. Adapters containing unique molecular indexes (UMIs) were ligated to gDNA fragments at room temperature for 30 min, and Sample Purification Beads were used to purify the fragments. Each library was labeled with a unique index via 15 cycles of amplification (98 °C for 10 s, 60 °C for 30 s and 72 °C for 30 s). The PCR products were hybridized with 5 µL probes at 57 °C for 16 h in hybridization buffer. Streptavidin Magnetic Beads were used to capture probes hybridized to the targeted library DNA regions of interest. After stringent washing with buffer EEW, the enriched DNA fragments were eluted from beads and subjected to a second round of hybridization-capture process. The enriched DNA fragments were amplified at 18 cycles (98 °C for 10 s, 60 °C for 30 s and 72 °C for 30 s). Sample Purification Beads were used to purify the final library; Qubit was used to measure concentration and a bioanalyzer was used to check the size distribution. Sequencing was done on the Nextseq 500 and sequence reads were mapped to the human reference genome (hg19) and analyzed using the TruSight Oncology 500 v2.2 Local App (Illumina).

Variant annotations were performed with ANNOVAR [37]. Identified variants with a population frequency of > 1% in 1000 Genomes Project were removed. Non-synonymous variants predicted to impact protein sequences derived from RefSeq transcripts and reported as pathogenic or likely pathogenic by at least three variant effect prediction tools (SIFT, FATHMM, LRT, MutationTaster, MutationAssessor, or PROVEAN), were retained, as were splice-site mutations within 2 bases of exon/intron boundaries. For small insertions and deletions, only changes resulting in frameshift mutations were retained. For *KIT*, non-frameshift mutations were validated by Sanger sequencing and retained from filtering applied to other genes to avoid losing known driver gain-of-function mutations. XenofilteR [38], a computational package designed to deconvolute sequence reads from different species, was used as a read-level quality-control and orthogonal verification step for variants identified by the TSO500 workflow and retained after ANNOVAR-based variant filtering. Briefly, unprocessed reads were aligned to the murine and human genomes separately and processed with XenofilteR to retain reads of human origin. Next, the genomic regions harboring candidate variants detected in PDXs but not present in the matched primary tumor were manually compared and examined in IGV [39]. Because the data were generated using a human-targeted capture panel, XenofilteR output was used for read-level verification to confirm that reads supporting the variant calls were retained after removal of reads classified as murine, rather than as a quantitative estimate of overall host contamination. Unfiltered variant calling results can be found at Additional File 1 archive.

### Data availability

The data generated in this study are available within the article and its additional data files. Raw NGS data has been deposited to the dbGaP database under accession number: phs004185.

### Statistical analysis

Pairwise Pearson correlations were used to compare genomic CNV profiles between samples. For the clustering of these profiles, segmentation data from each sample was collected and combined to a segment region by sample matrix, and data was then clustered with hierarchical clustering method using average Pearson correlation as the distance measure.

Tumor growth in xenograft studies was analyzed using a linear mixed effects model applied to log transformed tumor volumes. Zero values were imputed using one half of the smallest non-zero volume measured in the experiment. The model was fit using restricted maximum likelihood and included fixed effects for day, treatment group, and their interaction, with random intercepts and random slopes for each animal to account for repeated within-mouse measurements. Differences in tumor growth rates between Vehicle and Sunitinib were assessed by comparing model-estimated slopes. Day-specific treatment differences were estimated using model-derived marginal means, with pairwise contrasts evaluated using Wald tests and Holm adjustment for multiple comparisons. A significance threshold of *p* < 0.05 was used for all statistical assessments. The analyses were performed in R using lme4, lmerTest, and emmeans.

## Results

### GIST patient-derived xenografts preserve the histological, morphological, and molecular characteristics of the primary tumor

**Fig. 1:**
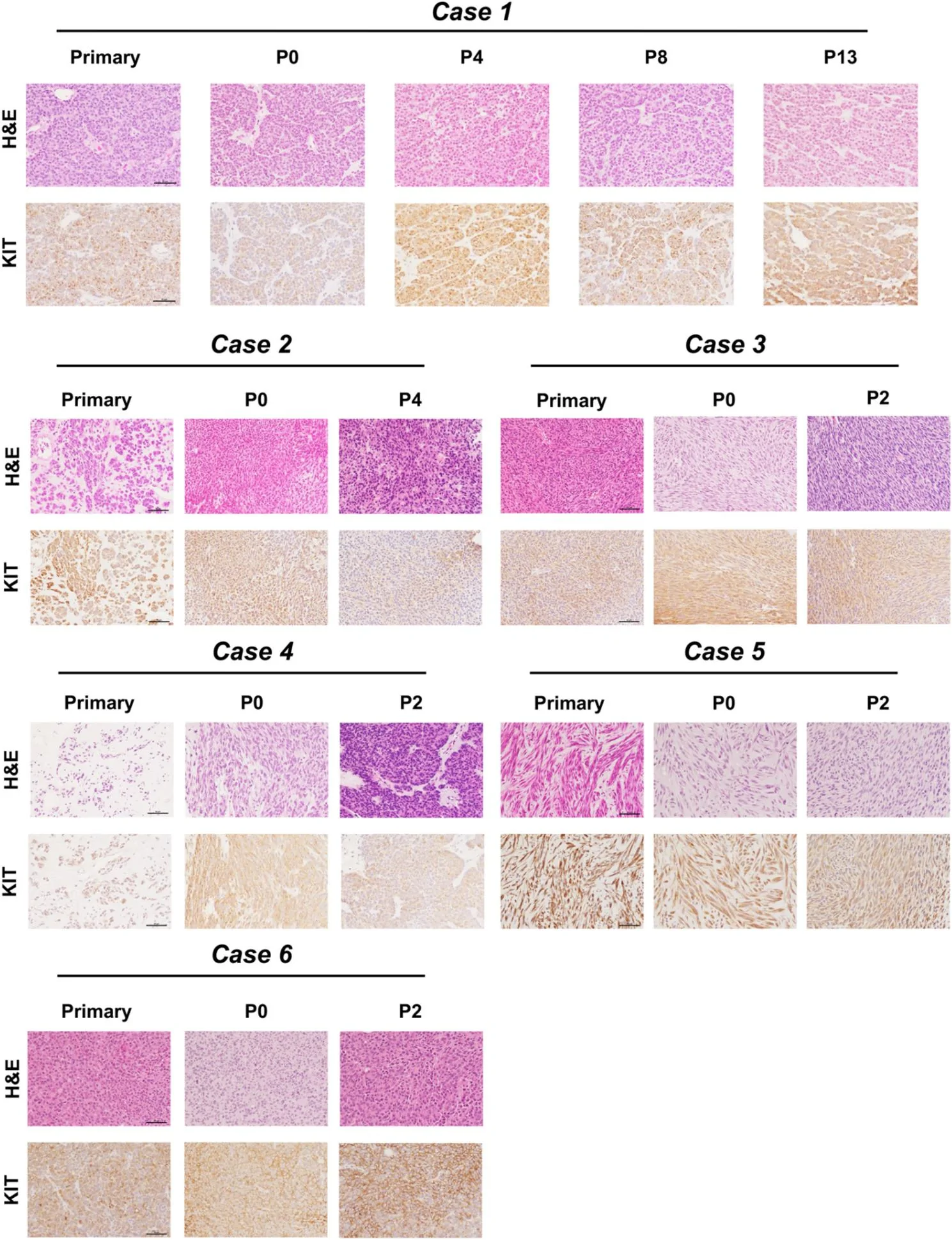
Representative hematoxylin/eosin section and KIT immunohistochemistry of patient primary tumors and patient-derived xenografts. Scale bar, 50 μm

Our laboratory has an ongoing interest in developing PDX mouse models from GIST of various genotypes for preclinical testing of novel therapeutic regimens. Using patient treatment status and tumor response as a guide to developing GIST PDX models, we have to date successfully engrafted and passaged 16/78 tumors into mice (success rate 20.5%). The only clinical feature that correlated with successful engraftment was metastasis, with 81.3% of these derived from metastatic tumors. For this study we chose six PDX models that were characterized through at least passage 2 (Table 1). These PDXs are derived from recurrent metastatic refractory GIST. The surgical specimens were obtained following failure of IM therapy in three cases and following 1–3 additional treatments in the other cases. We routinely genotype patient tumors by Sanger sequencing for primary mutations; these mutations (described in Table 1) were confirmed in all PDX passages by this approach as well as by targeted NGS, with the exception of the long deletion in case 5 that was not captured by the NGS platform (see below). The PDX models include two cases with identical exon 9 mutations (case 1,6) and four with various exon 11 mutations (cases 2–5). For all six cases the initial passage into mice (P0), and at least one later passage (e.g., P2, P4) were analyzed (Fig. 1). Two additional passages of case 1 (P8, P13), the first successfully engrafted tumor in our laboratory [27], were characterized. The PDX passages from these six cases maintain similar cellular morphology to the tumor of origin, as well as robust expression of KIT (Fig. 1) and DOG1 (Additional File 2). All established models have been cryopreserved at all passages, successfully thawed and re-engrafted, and will be available for sharing with the research community.

**Table 1:** Clinical characteristics of GIST cases used to establish PDXs

Case	KIT driver mutations^a^	Exon affected	PDX	Prior TKI(s)	Other therapies
1	c.1504_1509dup, p.(Ala502_Tyr503dup)	9	9.1	Imatinib Sunitinib Regorafenib	Radiation therapy Sirolimus
2	c.1670_1678del, p.(Trp557_Val559 delinsPhe)	11	11.2	Imatinib Sunitinib	Radiation therapy
3	c.1726 C > T, p.(Leu576Pro)	11	11.3	Imatinib	
4	c.1677_1712del, p.(Val560_Iso571del)	11	11.1	Imatinib	
5	c.1670_1714del, p.(Trp557_Asp572delinY)	11	11.4	Imatinib	
6	c.1504_1509dup, p.(Ala502_Tyr503dup)	9	9.2	Imatinib Sunitinib Avapritinib Regorafenib	Radiation therapy Sirolimus

^a^Accession NM_000222.2, NP000213.1

### Copy number variation (CNV) profiles of GIST PDXs mirror the tumor of origin

This study was initiated to examine the stability or evolution of GIST PDXs over time at the molecular genomic level. DNA from all tumors was subjected to both high-density genome-wide copy-number (CN) analysis and targeted next-generation sequencing. For CN analysis, genomic DNA from all 20 samples was analyzed using high-density Thermofisher CytoScan HD arrays. Probe hybridization data was processed using the Affymetrix GeneChip Command Console, and probeset data files from the AGCC were analyzed with the Bioconductor rCGH package [35]. Large-scale CN imbalances in the patient tumors identified by this pipeline are summarized in Additional File 3. All tumors exhibited genomic imbalances (mean = 11.0), with CN losses greater than gains (mean = 8.2, 2.8, respectively). Most of these patient tumors exhibit losses in chromosomes 1, 9, 10, 14, 15, and 22, while chromosome 8 gain is seen in 3 cases. This pattern of CN losses and gains in clinically advanced GIST has previously been described for larger GIST sample sets [40, 41].

**Fig. 2:**
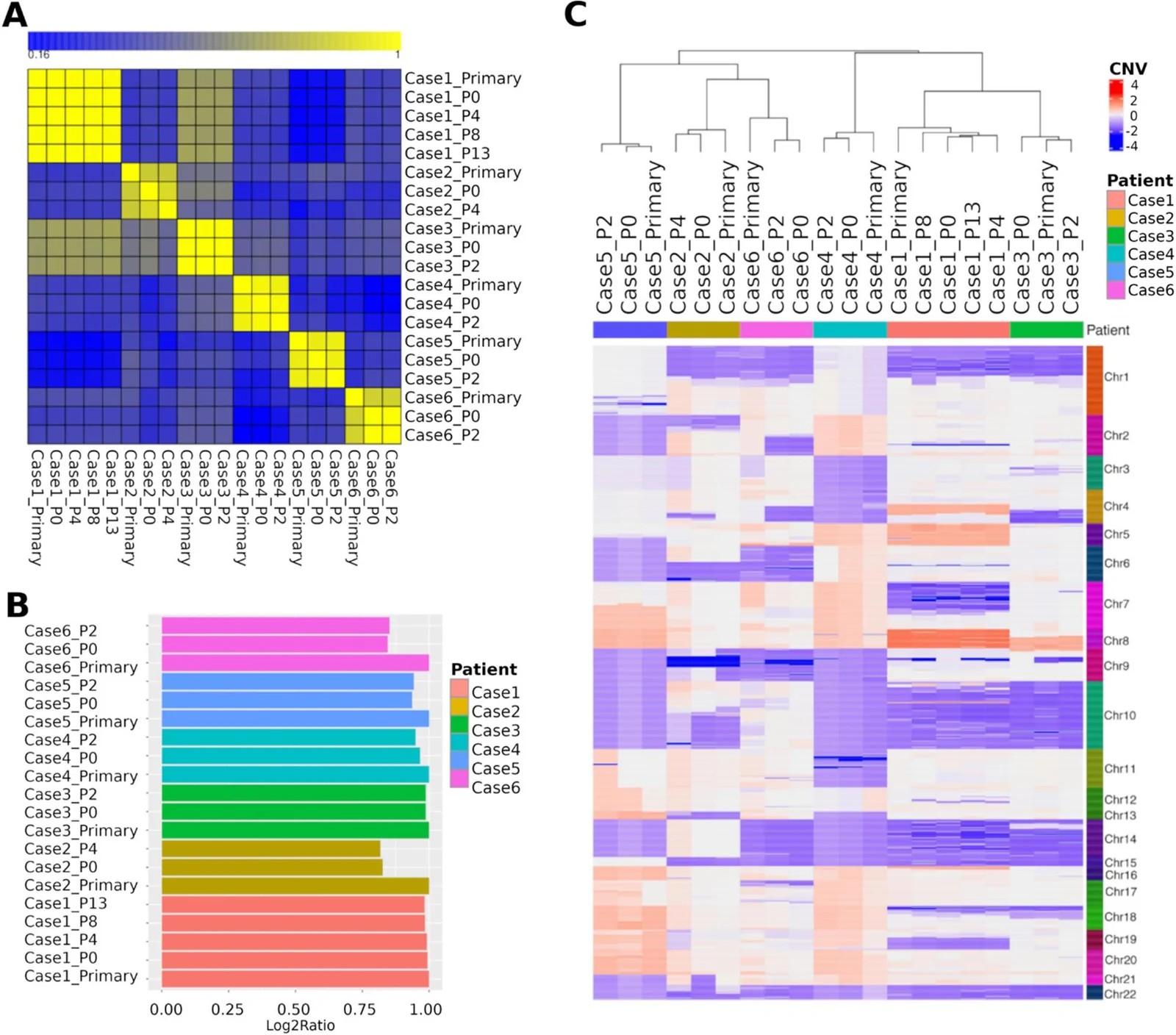
Fidelity of GIST PDX copy number profiles to parental tumors. **A** Correlation of patient and PDX sample copy number. Using the relative log ratio of all genes on the CytoScan HD arrays, pairwise Pearson correlations were calculated and plotted as color heatmap from yellow to blue (high to low correlation, respectively). **B** Correlation plots of PDX CNV profiles to primary samples. Bar graphs show the calculated paired Pearson correlation values (using relative log ratios) of each PDX to its corresponding tumor of origin. **C** Clustering of genome-wide CNV profiles of primary and PDX samples. Segmentation data from each sample was collected and combined to a segment region by sample matrix. Data was then clustered with hierarchical clustering method using average Pearson correlation as distance measure

The copy number profiles generated from our analysis were then compared across the sample set using statistical approaches. A pairwise comparison of all 20 samples, using the Pearson correlation coefficient as a measure, is shown as a heatmap in Fig. 2A. This analysis shows a high degree of correlation between PDX samples and their parental tumors, and much lower correlations between tumors from separate cases. Correlation values within cases range from ~ 0.82–0.99, while correlations across cases range from ~ 0.16–0.70 (Additional File 4). Figure 2B further illustrates the relative stability of PDX passages relative to parental tumors, which persists even out to 13 passages in case 1. The genome-wide CN profiles of the sample set, grouped by clustering using the average Pearson correlation distance measure, are shown in Fig. 2C. This data shows the consistency of genomic CNV within the 6 cases, with each PDX preferentially clustering with their corresponding parental tumors.

### Large-scale CNV changes are seen in several GIST PDX cases

**Fig. 3:**
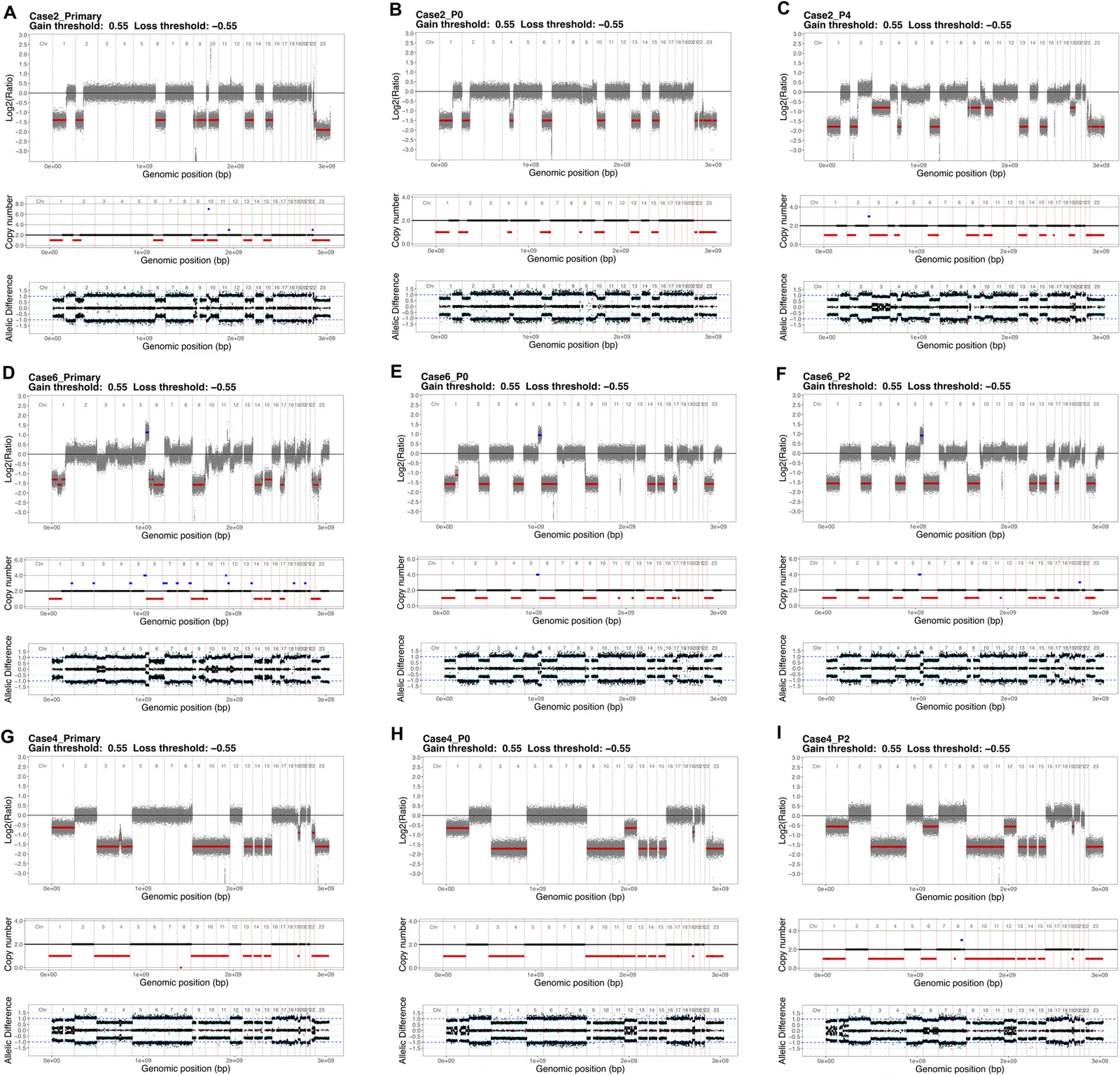
Genome-wide CN plots from the Bioconductor rCGH package for cases 2 (**A**-**C**), 6 (**D**-**F**), and 4 (**G**-**I**). Red and blue bars in the middle panels indicate regions of CN loss or gain called by the software

While the clustering analysis associates all GIST PDX passages to the same group as their primary tumors, the correlation values for cases 2 and 6 are notably lower than the others (Fig. 2B). The within-case Pearson correlations for tumors from case 2 (0.82) and from case 6 (0.84) are much lower than for the remaining cases (mean 0.97) (Additional File 4). From the CNV segmentation data generated by the Bioconductor package (Additional File 5) we calculated three measures of CNV prevalence: the number of CNV events, number of chromosomes affected by large-scale (> 5 Mb) CNVs, and total CNV size as a fraction of the genome (Additional File 6). The most highly correlated cases by the Pearson coefficient (cases 1 and 3) show strong conservation in all three measures, while case 5 shows a slight trend towards increased CNV events in both PDX passages. Surprisingly, both passages from case 4, which is fairly highly correlated by Pearson, show substantial increases in all three measures of CNV prevalence. For the less correlated cases, passage 4 from case 2 shows a substantial increase in all three measures over the primary tumor and initial passage. Both passages from case 6 show a decrease in CNV events and chromosome involvement, although the overall CNV fraction increases for the PDXs. To better understand the specific changes in these cases, we examined their genomic CN profiles on a case-by-case basis. Although the profiles from case 2 (Fig. 3A-C) show multiple shared regions of chromosome-level CN loss, additional losses not present in the primary tumor are seen in the two PDX passages (e.g. chromosome 21 in P0, chromosomes 3 and 19 in P4, part of chromosome 4 in both PDXs). The profiles from case 6 (Fig. 3D-F) also share similar overall patterns of chromosome loss and gain. However, both PDX samples exhibit CN loss across chromosome arms 2q and 4q that are not seen in the primary: these regions mainly account for increased CNV fraction in these samples. Closer inspection of the genomic CN profiles and segmentation tables identifies short regions of low-level CN gain in the case 6 primary (see middle panel of Fig. 3D); these events contribute to increased CNV events and chromosome involvement in this sample. Finally, P0 and P2 from case 4 both show low level CN loss across chromosome 12 not seen in the primary, while P2 alone shows additional CN loss across chromosome 6 (Fig. 3G-I). Both PDXs in this case show additional regions of low-level CN gain not called in the primary (Additional File 5). 

### Aberrations in cancer associated genes show preservation of the mutational landscape in PDXs

**Fig. 4:**
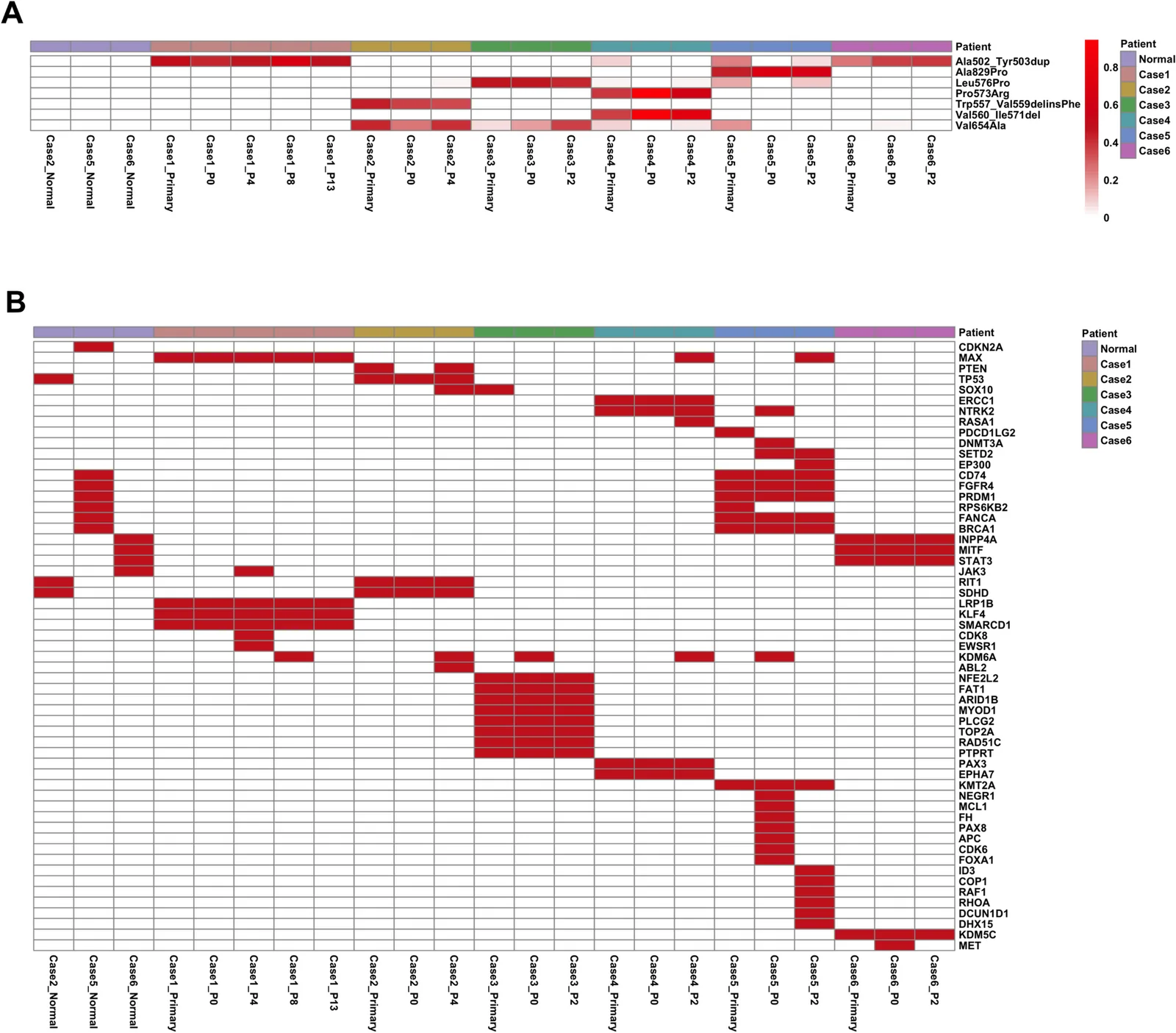
Deleterious variants in cancer associated genes were detected with targeted NGS using the Illumina TruSight Oncology 500 assay. **A** Gain of function mutations detected in KIT. Primary mutations identified by initial Sanger sequencing (Table 1) were detected at high AF by TSO500 with the exception of Trp557_Asp572delinsY (case 5), which is not detectable by NGS due to the size of the deletion (45 bp; c.1670_1714del). Protein-level mutation nomenclature used for labeling to match Table 1. Allele Frequency (AF) is indicated as fraction of calls (in scale 0 to 1) in shades of red. **B **Cancer associated genes harboring deleterious variants. NGS data were filtered by eliminating common variants identified by the 1000 Genomes Project, synonymous and non-deleterious variants

To further examine the stability of the PDX molecular landscape, DNA from primary tumors, PDXs, and normal tissues (cases 2, 5, 6 only) was subjected to targeted NGS profiling using the Illumina TruSight Oncology 500 assay. Non-deleterious variants were filtered from all specimens for the 523 gene analysis, with the exception of the *KIT* gene since non-frame shift mutations in *KIT* were not included based on our selection criteria. Primary mutations in *KIT* (Table 1) were detected at similar allelic frequencies (AF) in primary tumors and matched PDX tumors in cases 1–4 and 6 (Fig. 4A). The primary mutation in case 5, Trp557_Asp572delinsY, is a large deletion (45 bp; c.1670_1714del) which although detected by Sanger sequencing, was not detected by NGS. Short-read-based methods can fail to identify deletions of this size accurately [42].

Interestingly, while acquired *KIT* variants were detected in all cases driven by *KIT* exon 11 mutations (cases 2–5), no acquired mutations were detected in cases 1 and 6, the two cases with *KIT* exon 9 mutations. AFs for acquired mutations fluctuated between primary tumor and PDX passages. For instance, AF of Val654Ala in case 3 was 0.06 in primary tumor, 0.17 in P0, and increased to 0.38 in P2 (Fig. 4A**)**. Changes in prevalence of mutations between the primary tumor and PDX passages suggest initial polyclonality of GIST tumors and potential enrichment of sub-clonal populations through PDX passages. In cases 4 and 5, some acquired variants were lost in a similar manner, however, variants Leu576Pro and Ala502_Tyr503dup in case 5 were detected (with AF 0.15–0.20) in primary tumor and P2 (with AF 0.06–0.10), but not in P0 (Fig. 4A**)**, which suggests that variations in detection of mutations might result from single region sampling from heterogeneous tumors in some instances, rather than from any growth advantage [43]. The latter explanation might be supported by the observations that no loss of driver *KIT* mutations was observed in any case; while novel acquired *KIT* mutations, undetected in primary tumors, were detected at low levels in PDXs in the absence of selective TKI pressure.

Deleterious alterations in cancer associated genes are shown in Fig. 4B and Additional File 7 (gene and variant level representation, respectively). Some variants detected in tumor/PDX samples were also identified in their corresponding normal samples (see for instance case 6, Fig. 4B), suggesting that some germline variants pass through our filters. Therefore, it is possible that some mutations detected in cases 1, 3 and 4, where no matched normal control was available, are also germline variants. Changes in 43 genes in total (germline variants not included) were detected, with ~ 20 of these changes shared between primary tumors and matched PDXs. Three genes were altered in 2 out of 6 cases (*MAX*, *SOX10*, *NTRK2*). *De-novo* mutation of *KDM6A* was observed in varying passages in 5 out of 6 PDXs. Similar to inconsistencies in detection of *KIT* variants in case 5, as described above, alterations in other genes were also inconsistently detected between passages (case 2, *PTEN*; case 5 – group of 10 genes detected in P0 but not primary or P2). This is most likely due to variability in sampling from these clonal heterogeneous primary and PDX tumors, suggesting preservation of this polyclonal feature in PDXs **(**Fig. 4B**)**. Despite this, the overall pattern of mutations detected within cases, including primary tumors, PDXs and normal controls (where available), was remarkably similar. Additional File 7 demonstrates strong correlations between samples in each case, with all samples clustering together by case, confirming the high degree of similarity within the mutational landscape between primary tumor and matched PDX passages.

### In vivo testing of GIST PDXs

**Fig. 5:**
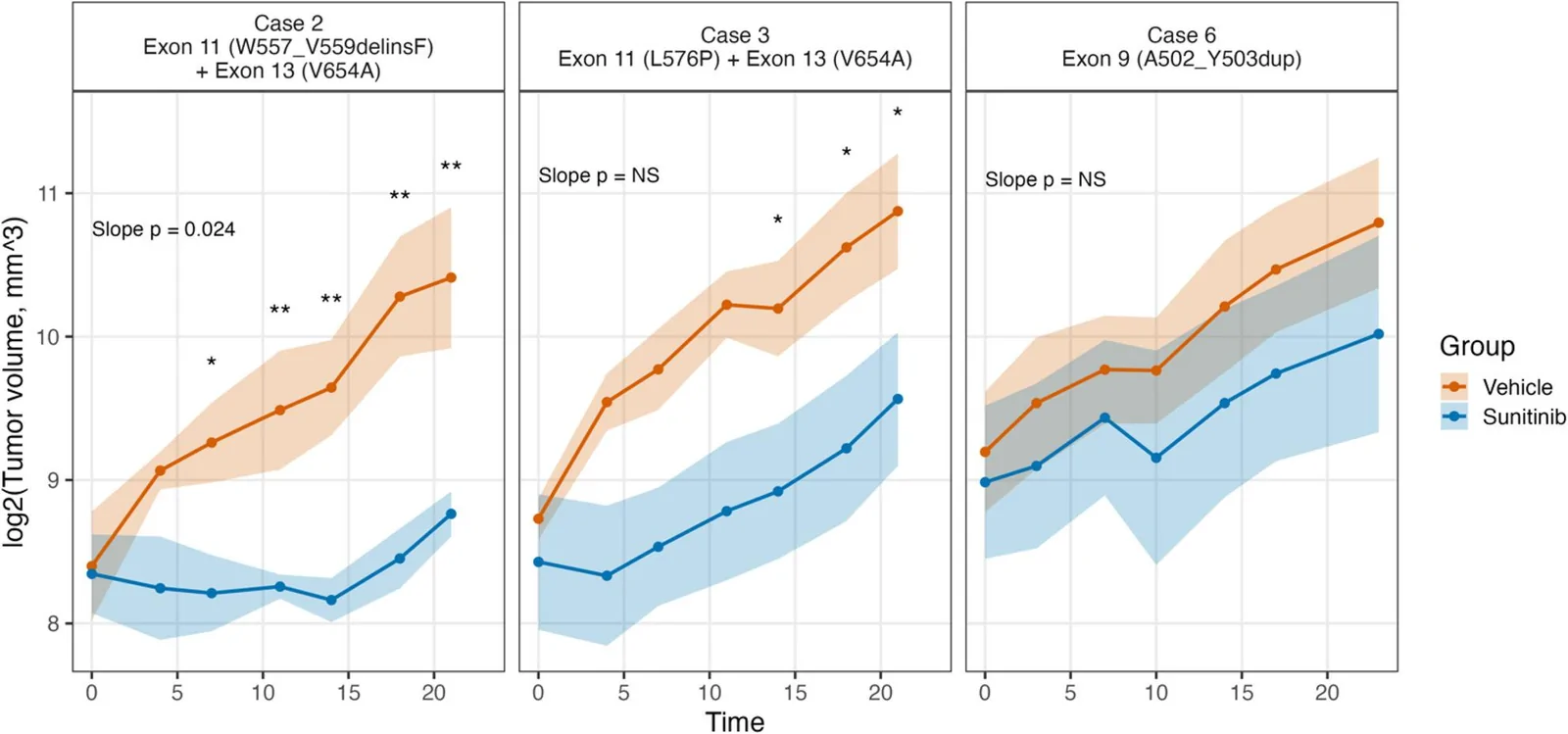
In vivo evaluation of PDX response to sunitinib treatment. Plots display log2-transformed mean tumor volumes (mm³) with standard error (SE). Statistical significance reflects differences in linear model estimated growth rates (slopes) on the log scale. Asterisks denote significant per-day comparisons between treatment groups (* *p* < 0.05, ** *p* < 0.01)

To assess whether these PDX models recapitulate clinically observed therapeutic responses, we evaluated tumor growth in vivo following treatment with the standard second-line therapy sunitinib. In the present study, we selected cases 2 and 3 for in vivo evaluation based on their similar genomic profiles, both harboring primary KIT exon 11 mutations and the acquired KIT exon 13 V654A mutation. In addition, case 6, carrying the KIT exon 9 mutation Ala502_Tyr503dup, was included as a genomically distinct model representative of an exon 9 mutant GIST. Case 1 representative of exon 9 mutant GIST was previously reported and demonstrated resistance to imatinib [27]. For each study, we randomized mice into two treatment groups: vehicle, and sunitinib **(**Fig. 5, A-C**)**. Tumor growth dynamics were evaluated using a linear mixed effects model applied to log transformed tumor volumes, allowing comparison of treatment-specific growth rates over time. Individual log-transformed tumor growth trajectories used for statistical analysis are shown in Additional File 8. Sunitinib treatment significantly reduced tumor growth rate compared with vehicle in case 2 (slope *p* = 0.024, Fig. 5). Consistent with this overall difference in growth rates, per-day comparisons indicated significant differences between treatment groups beginning at day 7 and continuing through day 21, the end of the study. In contrast, case 3 did not show a statistically significant difference in tumor growth rate between treatment groups (slope *p* = 0.133), although individual time points demonstrated significant differences at later time points (days 14–21). In Case 6, a modest reduction in tumor growth rate was observed (slope *p* = 0.047), while none of the individual day-wise comparisons reached statistical significance during the 23-day treatment period. The detailed results of the day-wise statistical comparisons between treatment groups are provided in Additional File 9.

## Discussion

Patients with advanced GIST who progress on approved tyrosine kinase inhibitors have limited remaining treatment options, underscoring the need for well-characterized preclinical models for translational research. Although GIST PDX models have previously been shown to preserve key features of the original tumors, including morphology and driver mutation status [19–30], recent studies in other cancer types have highlighted the importance of more extensive characterization to understand genomic changes that may occur during engraftment and passaging [14, 17, 18]. More recently, additional GIST PDX platforms have further demonstrated the translational utility of these models [30]. In this study, we molecularly characterized six GIST PDX models at the genome-wide level to evaluate their fidelity to matched patient tumors and their stability over serial passages.

We analyzed the genomic CNV profiles and mutational spectrum of multiple GIST PDX passages and compared them to matched patient tumors. The primary tumors for our study were collected from TKI-treated GIST patients. These PDXs maintained similar cellular morphology to the tumor of origin and exhibited the robust expression of KIT seen in parental tumors. CNV profiles of GIST PDXs closely mirrored the tumor of origin. Pairwise comparison of all 20 samples, using the Pearson correlation coefficient as a measure, demonstrated a high degree of correlation between PDX samples and their parental tumors, with much lower correlations between tumors from separate cases. Hierarchical clustering analysis assigned each PDX to a cluster with their cognate clinical samples. While the CNV analysis associates all GIST PDX passages with the same cluster as their primary tumor, the actual correlation values vary from case to case. We calculated the number of CNV events, the number of chromosomes affected by large-scale CNVs, and total CNV size as a fraction of the genome. Some models demonstrated a high level of conservation for these three measures (two cases), moderate to strong increase (three cases) and decrease in CNV events (one case) from the primary tumor and through passages. In some cases, such as in case 6 primary and case 4 PDX samples, these variations could be attributed to small differences in Log2 ratios that resulted in CNV segments being called in some samples and not in others. In other cases, several large regions of CN loss were clearly identified in PDX samples that were not detected in parental tumors. Regions of CN loss detected in PDXs but lacking in their parental tumors include part or all of chromosomes 2, 3, 4, 12, and 19. Interestingly, these regions of CN loss are not generally considered to be common in primary GIST [40, 41]. With the exception of the chromosome 4 losses seen in PDXs from both cases 2 and 6, these regions of *de novo* CN loss are also not shared across the cases in our small sample set. Overall, our CNV analysis suggests that GIST PDXs largely retain the genomic landscape of their parental tumors, while also revealing limited de novo CNV alterations in some models, most commonly involving regions of copy number loss. These findings are consistent with prior reports in other tumor types showing that PDX models can preserve core genomic architecture while undergoing limited clonal evolution during propagation [11, 17, 18].

Targeted NGS analysis of cancer-associated genes using the TSO 500 panel demonstrated preservation of the key features of the mutational landscape in GIST PDXs. First, looking at *KIT* variants, primary mutations in *KIT* were detected in tumors passaged through mice at similar AF to the primary tumors. Acquired *KIT* variants were detected in all TKI-treated cases driven by *KIT* exon 11 mutations, but none were detected in both cases possessing *KIT* exon 9 mutations, recapitulating the human clinical experience. However, unlike the primary mutations, the AFs for acquired *KIT* mutations fluctuated between primary tumor and PDX passages. In addition to fluctuations in allelic frequencies, we observed that some variants disappeared or were newly identified in PDXs compared to primary tumors. Similar differences have been previously reported in PDX models across multiple cancer types [30, 44–46]. These genomic differences are likely explained, at least in part, by baseline heterogeneity within the primary tumors [43, 47]. The region of the primary tumor sampled for establishing the PDX model might not include the entire representation of cell clones bearing individual genomic features and/or there is adaptation of passaged tumors to their new environment in a different species (mouse) which positively and negatively selects some sub-clonal populations [15, 44–46]. Taken together, these findings highlight the need for extensive characterization of PDX models to support their use in translational research.

Studies in other cancer types demonstrate that most cancer-driving mutations were retained between patient tumors and their subsequent passages; and therefore, unique mutations in PDXs might be considered as passenger mutations, which do not affect tumor biology [45, 48]. However, genomic evolution in PDX models can also occur at the level of large-scale structural changes, as DNA-based analyses have shown that copy number alterations may diverge substantially between primary tumors and their derived PDXs during engraftment and serial passaging [18]. Our findings are consistent with that conclusion. While all PDX tumors exhibited genomic imbalances, with CN losses greater than gains, based on our targeted NGS data, the mutational landscape of most common oncogenes was preserved between primary tumor and matched PDX passages. The clustering analysis, using Pearson correlation as distance measure, demonstrated that all samples clustered together by case. Interestingly, the same *de-novo* mutation in *KDM6A* (Ala806Thr) was observed in 5/6 (83%) of our models in at least 1 passage. KDM6A, a histone demethylase, has been shown to promote IM resistance in chronic myelogenous leukemia [49] and to be involved in the regulation of the tumor microenvironment in GIST [50]. KDM6A involvement in IM resistance may indicate selection of this mutation under TKI therapy, contributing to drug resistance in GIST. However, the emergence of these mutations could also reflect baseline subclonal heterogeneity, xenograft-driven selection or other technical factors. 50% of cases exhibited inactivating mutations in *MAX*. *MAX* inactivation has previously been shown by our group [51] and others [52, 53] to be a frequent event in GIST progression. Two additional genes were altered in 33% of cases: *SOX10* and *NTRK2*.

In addition to molecular characterization, we evaluated the in vivo response of selected PDX models to the standard second-line therapy sunitinib. Despite sharing similar KIT-driven genomic backgrounds, cases 2 and 3 demonstrated different responses to sunitinib in vivo. While case 2 showed a reduction in tumor growth rate, case 3 did not demonstrate a statistically significant difference in overall tumor growth, although differences at later time points were observed. Such variability is biologically plausible and does not reflect reduced model fidelity, as discordance between patient and xenograft drug responses has been reported in other PDX systems [15, 30, 44–46]. Instead, differential responses may result from co-occurring genomic alterations, differences in signaling pathway activity, intratumoral heterogeneity, or microenvironmental factors not captured by driver mutation status alone. Notably, targeted NGS identified additional non-KIT alterations in case 3, including mutations in FAT1 and NFE2L2, genes implicated in MAPK/ERK signaling and cellular stress responses, respectively, as well as dysregulation of TOP2A, all of which have been associated with TKI resistance in multiple cancer types [54–56]. Given the limited number of models examined, these findings should be considered preliminary. Evaluation of additional PDX models will be necessary to determine the biological factors driving differential sunitinib sensitivity. Case 6 showed no significant differences in growth rate with sunitinib treatment relative to vehicle in line with the lack of IM sensitivity previously demonstrated in case 1 [27]; both harbor the same KIT exon 9 duplication and were similarly pretreated with multiple TKIs.

Recent work in GIST has highlighted the role of tumor microenvironment and metabolic adaptation in TKI resistance, including cancer-associated fibroblast-mediated metabolic reprogramming [57]. Additional studies have demonstrated that the tumor microenvironment can influence PDX behavior through non-genetic mechanisms including pathway rewiring, metabolic adaptation, and cellular stress-response programs [17]. Emerging multi-omic analyses have further elaborated on the complexity of tumor evolution; for example, proteomic profiling of GIST has identified signaling heterogeneity associated with differential imatinib sensitivity beyond KIT/PDGFRA genotype alone, consistent with the genotype-phenotype discordance observed in our in vivo drug response data [31]. Taken together, our findings, along with emerging evidence from complementary approaches, support the use of deeply characterized PDX models as translational tools and underscore that driver mutation status alone, while foundational, represents only one layer of a multi-omic framework needed to fully predict therapeutic response.

Although numerous GIST PDXs have been established by other investigators and shown to preserve cellular morphology and KIT status, detailed genome-wide molecular characterization remains limited. Our study adds integrated CNV and targeted mutational profiling across serial passages, complementing recently published GIST PDX platforms and extending the available molecular characterization of these models. Our results confirm what has been seen in other tumor types: strong sample correlations, with individual changes in CNV and mutational spectrum. However, there are limitations to our current study. The first limitation is the limited sample size related to the availability of tumor specimens due to GIST being a rare cancer. There have been only a few reports of PDX models for GIST. The success rate of PDX establishment in this study was 20.6% (which is similar to the previously reported rate of 17% [24] or 13.5% in [30]. Second, one of the reported issues of using PDX models is the potential for contamination of human tumor cells with mouse stroma [58]. In this study, we used a targeted NGS approach which limited our ability to quantify the degree of murine cells and measure changes over multiple passages. Third, our study was not focused on changes in signaling transduction over passages. It has been shown that differences in gene expression and activity of signaling kinases might appear as a result of different environments in cell and PDX models [59–61]. Differential gene expression and/or alterations in signaling pathways might change growth programs in these models and may be interconnected with tumors’ response to TKI therapy. Understanding the functional consequences of the specific genomic changes identified here will require larger cohorts and additional complementary platforms, including transcriptomic, kinome, proteomic, and chromatin-level profiling. Finally, while establishing PDX models, engraftment bias remains a potential limitation. Successfully engrafted tumors may not fully capture the cellular heterogeneity of the original specimen and may be enriched for highly proliferative, genomically aggressive subclones with a growth advantage in mice [62]. Therefore, while our PDX models retain the key histopathologic and genomic features of the parental tumors, they may not fully represent the complete biological complexity of TKI-refractory GIST.

In summary, we have molecularly characterized six GIST PDX models with diverse treatment histories and genotypes derived from clinically refractory tumors, an area of substantial unmet need in GIST research. Motivated by recent reports of PDX evolution in other tumor types [7–14, 63, 64], we evaluated these models at the levels of genome-wide copy number and cancer-associated gene alterations across serial passages. Our results show that these GIST PDX models preserve the key molecular features of their corresponding primary tumors while also capturing limited genomic variation consistent with baseline tumor heterogeneity and possible clonal selection during propagation. By combining deep molecular characterization with functional in vivo testing, this study expands the available repertoire of preclinical GIST models by adding six well-characterized GIST PDX models as valuable resources for translational studies of clinically refractory disease, therapeutic response, and resistance mechanisms.

## Supplementary Information

Supplementary Material 1.

Supplementary Material 2.

Supplementary Material 3.

Supplementary Material 4.

Supplementary Material 5.

Supplementary Material 6.

Supplementary Material 7.

Supplementary Material 8.

Supplementary Material 9.

## Data Availability

The data generated in this study are available within the article and its additional data files. Raw NGS data has been deposited to the dbGaP database under accession number: phs004185.

## Abbreviations

CN: Copy number
CNV: Copy number variation
DOG1: Discovered on GIST-1
GIST: Gastrointestinal Stromal Tumor
H&E: Hematoxylin and Eosin
IM: Imatinib mesylate
LRR: Log_2_ relative ratio
NGS: Next-generation sequencing
NSG: NOD scid gamma
PCR: Polymerase chain reaction
PDX: Patient-derived xenograft
PDGFRA: Platelet-derived growth factor alpha
TKI: Tyrosine kinase inhibitor

## References

[1] Goettsch WG, Bos SD, Breekveldt-Postma N, Casparie M, Herings RM, Hogendoorn PC. Incidence of gastrointestinal stromal tumours is underestimated: results of a nation-wide study. Eur J Cancer. 2005;41(18):2868–72. 10.1016/j.ejca.2005.09.009 16293410

[2] Corless CL, Barnett CM, Heinrich MC. Gastrointestinal stromal tumours: origin and molecular oncology. Nat Rev Cancer. 2011;11(12):865–78. 10.1038/nrc3143 22089421

[3] Hirota S, Isozaki K, Moriyama Y, Hashimoto K, Nishida T, Ishiguro S, Kawano K, Hanada M, Kurata A, Takeda M, et al. Gain-of-function mutations of c-kit in human gastrointestinal stromal tumors. Science. 1998;279(5350):577–80. 10.1126/science.279.5350.577 9438854

[4] Heinrich MC, Corless CL, Duensing A, McGreevey L, Chen CJ, Joseph N, Singer S, Griffith DJ, Haley A, Town A, et al. PDGFRA activating mutations in gastrointestinal stromal tumors. Science. 2003;299(5607):708–10. 10.1126/science.1079666 12522257

[5] Rivera M, Fichtner I, Wulf-Goldenberg A, Sers C, Merk J, Patone G, Alp KM, Kanashova T, Mertins P, Hoffmann J, et al. Patient-derived xenograft (PDX) models of colorectal carcinoma (CRC) as a platform for chemosensitivity and biomarker analysis in personalized medicine. Neoplasia. 2021;23(1):21–35. 10.1016/j.neo.2020.11.005 PMC7680704 33212364

[6] Kopetz S, Lemos R, Powis G. The promise of patient-derived xenografts: the best laid plans of mice and men. Clin Cancer Res. 2012;18(19):5160–2. 10.1158/1078-0432.CCR-12-2408 PMC4217576 22912394

[7] Jung HY, Kim TH, Lee JE, Kim HK, Cho JH, Choi YS, Shin S, Lee SH, Rhee H, Lee HK, et al. PDX models of human lung squamous cell carcinoma: consideration of factors in preclinical and co-clinical applications. J Transl Med. 2020;18(1):307. 10.1186/s12967-020-02473-y PMC7409653 32762722

[8] Kim K, Hu W, Audenet F, Almassi N, Hanrahan AJ, Murray K, Bagrodia A, Wong N, Clinton TN, Dason S, et al. Modeling biological and genetic diversity in upper tract urothelial carcinoma with patient derived xenografts. Nat Commun. 2020;11(1):1975. 10.1038/s41467-020-15885-7 PMC7181640 32332851

[9] Julien S, Merino-Trigo A, Lacroix L, Pocard M, Goere D, Mariani P, Landron S, Bigot L, Nemati F, Dartigues P, et al. Characterization of a large panel of patient-derived tumor xenografts representing the clinical heterogeneity of human colorectal cancer. Clin Cancer Res. 2012;18(19):5314–28. 10.1158/1078-0432.CCR-12-0372 22825584

[10] Cho YB, Hong HK, Choi YL, Oh E, Joo KM, Jin J, Nam DH, Ko YH, Lee WY. Colorectal cancer patient-derived xenografted tumors maintain characteristic features of the original tumors. J Surg Res. 2014;187(2):502–9. 10.1016/j.jss.2013.11.010 24332554

[11] Wang D, Pham NA, Tong J, Sakashita S, Allo G, Kim L, Yanagawa N, Raghavan V, Wei Y, To C, et al. Molecular heterogeneity of non-small cell lung carcinoma patient-derived xenografts closely reflect their primary tumors. Int J Cancer. 2017;140(3):662–73. 10.1002/ijc.30472 27750381

[12] Cancer Genome Atlas Research N. Comprehensive molecular characterization of clear cell renal cell carcinoma. Nature. 2013;499(7456):43–9. 10.1038/nature12222 PMC3771322 23792563

[13] Li H, Zhu Y, Tang X, Li J, Li Y, Zhong Z, Ding G, Li Y. Integrated analysis of transcriptome in cancer patient-derived xenografts. PLoS ONE. 2015;10(5):e0124780. 10.1371/journal.pone.0124780 PMC4423839 25951608

[14] Shi J, Li Y, Jia R, Fan X. The fidelity of cancer cells in PDX models: Characteristics, mechanism and clinical significance. Int J Cancer. 2020;146(8):2078–88. 10.1002/ijc.32662 31479514

[15] Hynds RE, Huebner A, Pearce DR, Hill MS, Akarca AU, Moore DA, Ward S, Gowers K, Karasaki T, Bakir MA, et al. Representation of genomic intratumor heterogeneity in multi-region non-small cell lung cancer patient-derived xenograft models. Nat Commun. 2024;15:4653. 10.1038/s41467-024-47547-3 PMC11143323 38821942

[16] Yuan Y, Gao H, Li Y, Jiao X. The latest progress of personalized drug screening and therapy research for common clinical tumors through the PDX model platform. J Pharm Anal. 2025;15(10):101225. 10.1016/j.jpha.2025.101225 PMC12616016 41245660

[17] Ben-David U, Ha G, Tseng YY, Greenwald NF, Oh C, Shih J, McFarland JM, Wong B, Boehm JS, Beroukhim R, et al. Patient-derived xenografts undergo mouse-specific tumor evolution. Nat Genet. 2017;49(11):1567–75. 10.1038/ng.3967 PMC5659952 28991255

[18] Hoge ACH, Getz M, Zimmer A, Ko M, Raz L, Beroukhim R, Golub TR, Ha G, Ben-David U. DNA-based copy number analysis confirms genomic evolution of PDX models. Npj Precis Onc. 2021;6(1):30. 10.1038/s41698-022-00268-6 PMC9050710 35484194

[19] Wang Y, Wozniak A, Wellens J, Gebreyohannes YK, Guillen MJ, Aviles PM, Debiec-Rychter M, Sciot R, Schoffski P. Plocabulin, a novel tubulin inhibitor, has potent antitumor activity in patient-derived xenograft models of gastrointestinal stromal tumors. Transl Oncol. 2020;13(11):100832. 10.1016/j.tranon.2020.100832 PMC7381700 32711367

[20] Van Looy T, Gebreyohannes YK, Wozniak A, Cornillie J, Wellens J, Li H, Vanleeuw U, Floris G, Debiec-Rychter M, Sciot R, et al. Characterization and assessment of the sensitivity and resistance of a newly established human gastrointestinal stromal tumour xenograft model to treatment with tyrosine kinase inhibitors. Clin Sarcoma Res. 2014;4:10. 10.1186/2045-3329-4-10 PMC4134663 25132955

[21] Sicklick JK, Leonard SY, Babicky ML, Tang CM, Mose ES, French RP, Jaquish DV, Hoh CK, Peterson M, Schwab R, et al. Generation of orthotopic patient-derived xenografts from gastrointestinal stromal tumor. J Transl Med. 2014;12:41. 10.1186/1479-5876-12-41 PMC3930355 24507750

[22] Huynh H, Lee JW, Chow PK, Ngo VC, Lew GB, Lam IW, Ong HS, Chung A, Soo KC. Sorafenib induces growth suppression in mouse models of gastrointestinal stromal tumor. Mol Cancer Ther. 2009;8(1):152–9. 10.1158/1535-7163.MCT-08-0553 19139124

[23] Revheim ME, Seierstad T, Berner JM, Bruland OS, Roe K, Ohnstad HO, Bjerkehagen B, Bach-Gansmo T. Establishment and characterization of a human gastrointestinal stromal tumour (GIST) xenograft in athymic nude mice. Anticancer Res. 2009;29(11):4331–6. 20032375

[24] Na YS, Ryu MH, Park YS, Lee CW, Lee JK, Park Y, Park JM, Ma J, Kang YK. Establishment of patient-derived xenografts from patients with gastrointestinal stromal tumors: analysis of clinicopathological characteristics related to engraftment success. Sci Rep. 2020;10(1):7996. 10.1038/s41598-020-64552-w PMC7224375 32409663

[25] Floris G, Wozniak A, Sciot R, Li H, Friedman L, Van Looy T, Wellens J, Vermaelen P, Deroose CM, Fletcher JA, et al. A potent combination of the novel PI3K Inhibitor, GDC-0941, with imatinib in gastrointestinal stromal tumor xenografts: long-lasting responses after treatment withdrawal. Clin Cancer Res. 2013;19(3):620–30. 10.1158/1078-0432.CCR-12-2853 PMC4405777 23231951

[26] Ye S, Sharipova D, Kozinova M, Klug L, D’Souza J, Belinsky MG, Johnson KJ, Einarson MB, Devarajan K, Zhou Y, et al. Identification of Wee1 as a target in combination with avapritinib for gastrointestinal stromal tumor treatment. JCI Insight. 2021;6(2):143474. 10.1172/jci.insight.143474 PMC7934848 33320833

[27] Kozinova M, Joshi S, Ye S, Belinsky MG, Sharipova D, Farma JM, Reddy SS, Litwin S, Devarajan K, Campos AR et al. Combined Inhibition of AKT and KIT Restores Expression of Programmed Cell Death 4 (PDCD4) in Gastrointestinal Stromal Tumor. *Cancers (Basel)* 2021, 13(15). 10.3390/cancers13153699 PMC8345102 34359600

[28] Yebra M, Bhargava S, Kumar A, Burgoyne AM, Tang CM, Yoon H, Banerjee S, Aguilera J, Cordes T, Sheth V, et al. Establishment of Patient-Derived Succinate Dehydrogenase-Deficient Gastrointestinal Stromal Tumor Models for Predicting Therapeutic Response. Clin Cancer Res. 2022;28(1):187–200. 10.1158/1078-0432.CCR-21-2092 PMC8738129 34426440

[29] Flavahan WA, Drier Y, Johnstone SE, Hemming ML, Tarjan DR, Hegazi E, Shareef SJ, Javed NM, Raut CP, Eschle BK, et al. Altered chromosomal topology drives oncogenic programs in SDH-deficient GISTs. Nature. 2019;575(7781):229–33. 10.1038/s41586-019-1668-3 PMC6913936 31666694

[30] De Sutter L, De Cock L, Wang C, Gorgels D, Wyns K, Verbeeck K, Vanleeuw U, Douchy T, Hompes D, Jaekers J, et al. Patient-derived xenograft models of gastrointestinal stromal tumors provide a ready-to-use platform for translational research. Dis Model Mech. 2025;18(2):052225. 10.1242/dmm.052225 PMC11876840 39853155

[31] Sun M, Tong Y, Yuan W, Wang Y, Pu Y, Huang W, Lv B, Xu C, Jiang W, Luo R et al. Proteomic Characterization Identifies Clinically Relevant Subgroups of Gastrointestinal Stromal Tumors. *Gastroenterology* 2024, (166)3:450–465. 10.1053/j.gastro.2023.11.284 37995868

[32] Pestinger V, Smith M, Sillo T, Findlay JM, Laes JF, Martin G, Middleton G, Taniere P, Beggs AD. Use of an Integrated Pan-Cancer Oncology Enrichment Next-Generation Sequencing Assay to Measure Tumour Mutational Burden and Detect Clinically Actionable Variants. Mol Diagn Ther. 2020;24(3):339–49. 10.1007/s40291-020-00462-x PMC7264086 32306292

[33] Belinsky MG, Rink L, Flieder DB, Jahromi MS, Schiffman JD, Godwin AK, Mehren M. Overexpression of insulin-like growth factor 1 receptor and frequent mutational inactivation of SDHA in wild-type SDHB-negative gastrointestinal stromal tumors. Genes Chromosomes Cancer. 2013;52(2):214–24. 10.1002/gcc.22023 PMC3564228 23109135

[34] Belinsky MG, Cai KQ, Zhou Y, Luo B, Pei J, Rink L, von Mehren M. Succinate dehydrogenase deficiency in a PDGFRA mutated GIST. BMC Cancer. 2017;17(1):512. 10.1186/s12885-017-3499-7 PMC5541693 28768491

[35] Commo F, Guinney J, Ferte C, Bot B, Lefebvre C, Soria JC, Andre F. rCGH: a comprehensive array-based genomic profile platform for precision medicine. Bioinformatics. 2016;32(9):1402–4. 10.1093/bioinformatics/btv718 PMC4848396 26708336

[36] Venkatraman ES, Olshen AB. A faster circular binary segmentation algorithm for the analysis of array CGH data. Bioinformatics. 2007;23(6):657–63. 10.1093/bioinformatics/btl646 17234643

[37] Wang K, Li M, Hakonarson H. ANNOVAR: functional annotation of genetic variants from high-throughput sequencing data. Nucleic Acids Res. 2010;38(16):e164. 10.1093/nar/gkq603 PMC2938201 20601685

[38] Kluin RJC, Kemper K, Kuilman T, de Ruiter JR, Iyer V, Forment JV, Cornelissen-Steijger P, de Rink I, Ter Brugge P, Song JY, et al. XenofilteR: computational deconvolution of mouse and human reads in tumor xenograft sequence data. BMC Bioinformatics. 2018;19(1):366. 10.1186/s12859-018-2353-5 PMC6172735 30286710

[39] Robinson JT, Thorvaldsdottir H, Wenger AM, Zehir A, Mesirov JP. Variant Review with the Integrative Genomics Viewer. Cancer Res. 2017;77(21):e31–4. 10.1158/0008-5472.CAN-17-0337 PMC5678989 29092934

[40] Gunawan B, von Heydebreck A, Sander B, Schulten HJ, Haller F, Langer C, Armbrust T, Bollmann M, Gasparov S, Kovac D, et al. An oncogenetic tree model in gastrointestinal stromal tumours (GISTs) identifies different pathways of cytogenetic evolution with prognostic implications. J Pathol. 2007;211(4):463–70. 10.1002/path.2128 17226762

[41] Wozniak A, Sciot R, Guillou L, Pauwels P, Wasag B, Stul M, Vermeesch JR, Vandenberghe P, Limon J, Debiec-Rychter M. Array CGH analysis in primary gastrointestinal stromal tumors: cytogenetic profile correlates with anatomic site and tumor aggressiveness, irrespective of mutational status. Genes Chromosomes Cancer. 2007;46(3):261–76. 10.1002/gcc.20408 17171690

[42] Mahmoud M, Gobet N, Cruz-Davalos DI, Mounier N, Dessimoz C, Sedlazeck FJ. Structural variant calling: the long and the short of it. Genome Biol. 2019;20(1):246. 10.1186/s13059-019-1828-7 PMC6868818 31747936

[43] Gerlinger M, Rowan AJ, Horswell S, Math M, Larkin J, Endesfelder D, Gronroos E, Martinez P, Matthews N, Stewart A, et al. Intratumor heterogeneity and branched evolution revealed by multiregion sequencing. N Engl J Med. 2012;366(10):883–92. 10.1056/NEJMoa1113205 PMC4878653 22397650

[44] Choi YY, Lee JE, Kim H, Sim MH, Kim KK, Lee G, Kim HI, An JY, Hyung WJ, Kim CB, et al. Establishment and characterisation of patient-derived xenografts as paraclinical models for gastric cancer. Sci Rep. 2016;6:22172. 10.1038/srep22172 PMC4772087 26926953

[45] Hao C, Wang L, Peng S, Cao M, Li H, Hu J, Huang X, Liu W, Zhang H, Wu S, et al. Gene mutations in primary tumors and corresponding patient-derived xenografts derived from non-small cell lung cancer. Cancer Lett. 2015;357(1):179–85. 10.1016/j.canlet.2014.11.024 PMC4301580 25444907

[46] Villafranca-Magdalena B, Masferrer-Ferragutcasas C, Lopez-Gil C, Coll-de la Rubia E, Rebull M, Parra G, Garcia A, Reques A, Cabrera S, Colas E, et al. Genomic Validation of Endometrial Cancer Patient-Derived Xenograft Models as a Preclinical Tool. Int J Mol Sci. 2022;23(11):6266. 10.3390/ijms23116266 PMC9181722 35682944

[47] Cassidy JW, Batra AS, Greenwood W, Bruna A. Patient-derived tumour xenografts for breast cancer drug discovery. Endocr Relat Cancer. 2016;23(12):T259–70. 10.1530/ERC-16-0251 PMC5118939 27702751

[48] Depreeuw J, Hermans E, Schrauwen S, Annibali D, Coenegrachts L, Thomas D, Luyckx M, Gutierrez-Roelens I, Debruyne D, Konings K, et al. Characterization of patient-derived tumor xenograft models of endometrial cancer for preclinical evaluation of targeted therapies. Gynecol Oncol. 2015;139(1):118–26. 10.1016/j.ygyno.2015.07.104 26232337

[49] Zhang C, Shen L, Zhu Y, Xu R, Deng Z, Liu X, Ding Y, Wang C, Shi Y, Bei L, et al. KDM6A promotes imatinib resistance through YY1-mediated transcriptional upregulation of TRKA independently of its demethylase activity in chronic myelogenous leukemia. Theranostics. 2021;11(6):2691–705. 10.7150/thno.50571 PMC7806474 33456567

[50] Shen C, Han L, Liu B, Zhang G, Cai Z, Yin X, Yin Y, Chen Z, Zhang B. The KDM6A-SPARCL1 axis blocks metastasis and regulates the tumour microenvironment of gastrointestinal stromal tumours by inhibiting the nuclear translocation of p65. Br J Cancer. 2022;126(10):1457–69. 10.1038/s41416-022-01728-3 PMC9090789 35136209

[51] Belinsky MG, Rink L, Cai KQ, Capuzzi SJ, Hoang Y, Chien J, Godwin AK, von Mehren M. Somatic loss of function mutations in neurofibromin 1 and MYC associated factor X genes identified by exome-wide sequencing in a wild-type GIST case. BMC Cancer. 2015;15:887. 10.1186/s12885-015-1872-y PMC4641358 26555092

[52] Pantaleo MA, Urbini M, Indio V, Ravegnini G, Nannini M, De Luca M, Tarantino G, Angelini S, Gronchi A, Vincenzi B, et al. Genome-Wide Analysis Identifies MEN1 and MAX Mutations and a Neuroendocrine-Like Molecular Heterogeneity in Quadruple WT GIST. Mol Cancer Res. 2017;15(5):553–62. 10.1158/1541-7786.MCR-16-0376 28130400

[53] Schaefer IM, Wang Y, Liang CW, Bahri N, Quattrone A, Doyle L, Marino-Enriquez A, Lauria A, Zhu M, Debiec-Rychter M, et al. MAX inactivation is an early event in GIST development that regulates p16 and cell proliferation. Nat Commun. 2017;8:14674. 10.1038/ncomms14674 PMC5344969 28270683

[54] Chen ZG, Saba NF, Teng Y. The diverse functions of FAT1 in cancer progression: good, bad, or ugly? J Exp Clin Cancer Res. 2022;41(1):248. 10.1186/s13046-022-02461-8 PMC9377080 35965328

[55] Hellyer JA, Stehr H, Das M, Padda SK, Ramchandran K, Neal JW, Diehn M, Wakelee HA. Impact of KEAP1/NFE2L2/CUL3 mutations on duration of response to EGFR tyrosine kinase inhibitors in EGFR mutated non-small cell lung cancer. Lung Cancer. 2019;134:42–5. 10.1016/j.lungcan.2019.05.002 31319993

[56] Wang Z, Zhu Q, Li X, Ren X, Li J, Zhang Y, Zeng S, Xu L, Dong X, Zhai B. TOP2A inhibition reverses drug resistance of hepatocellular carcinoma to regorafenib. Am J Cancer Res. 2022;12(9):4343–60. PMC9548008 36225636

[57] Li C, Teng T, Lin X, Sun X, Yi T, Wang H, Gao X, Shen K. Cancer-Associated Fibroblasts Promote Imatinib Resistance in Gastrointestinal Stromal Tumors through PGK1-Mediated Metabolic Reprogramming. Cancer Res. 2026;16(4):988–1009. 10.1158/0008-5472.CAN-25-1404 41270149

[58] Yoshida GJ. Applications of patient-derived tumor xenograft models and tumor organoids. J Hematol Oncol. 2020;13(1):4. 10.1186/s13045-019-0829-z PMC6947974 31910904

[59] Coleman O, Henry M, O’Neill F, Roche S, Swan N, Boyle L, Murphy J, Meiller J, Conlon NT, Geoghegan J, et al. A Comparative Quantitative LC-MS/MS Profiling Analysis of Human Pancreatic Adenocarcinoma, Adjacent-Normal Tissue, and Patient-Derived Tumour Xenografts. Proteomes. 2018;6(4):45. 10.3390/proteomes6040045 PMC6313850 30404163

[60] Kanaya N, Somlo G, Wu J, Frankel P, Kai M, Liu X, Wu SV, Nguyen D, Chan N, Hsieh MY, et al. Characterization of patient-derived tumor xenografts (PDXs) as models for estrogen receptor positive (ER+HER2- and ER+HER2+) breast cancers. J Steroid Biochem Mol Biol. 2017;170:65–74. 10.1016/j.jsbmb.2016.05.001 PMC5094906 27154416

[61] Martinez R. 3rd, Huang W, Buck H, Rea S, Defnet AE, Kane MA, Shapiro P. Proteomic Changes in the Monolayer and Spheroid Melanoma Cell Models of Acquired Resistance to BRAF and MEK1/2 Inhibitors. ACS Omega. 2022;7(4):3293–311. 10.1021/acsomega.1c05361 PMC8811929 35128241

[62] Cassidy JW, Caldas C, Bruna A. Maintaining Tumor Heterogeneity in Patient-Derived Tumor Xenografts. Cancer Res. 2015;75(15):2963–8. 10.1158/0008-5472.CAN-15-0727 PMC4539570 26180079

[63] Donhuijsen K, Budach V, van Beuningen D, Schmidt U. Instability of xenotransplanted soft tissue sarcomas. Morphologic and flow cytometric results. Cancer. 1988;61(1):68–75. 10.1002/1097-0142(19880101)61:1<68::aid-cncr2820610112>3.0.co;2-e 3334953

[64] Yamaguchi R, Perkins G. Animal models for studying tumor microenvironment (TME) and resistance to lymphocytic infiltration. Cancer Biol Ther. 2018;19(9):745–54. 10.1080/15384047.2018.1470722 PMC6154837 29723108

