# Supplementary material for: Establishment and genomic validation of novel patient-derived xenograft models for drug discovery in gastrointestinal stromal tumor
Source: BMC Cancer. 2026 Aug 10;26:1213. doi: 10.1186/s12885-026-16659-y (PMC13654211; doi:10.1186/s12885-026-16659-y)
Supplement: Supplementary file 1 [file 12885_2026_16659_MOESM2_ESM.docx]

**Additional File 2**. Representative DOG1 immunohistochemistry of patient primary tumors and patient-derived xenografts. Scale bar, 50 mm.
