# Supplementary material for: Establishment and genomic validation of novel patient-derived xenograft models for drug discovery in gastrointestinal stromal tumor
Source: BMC Cancer. 2026 Aug 10;26:1213. doi: 10.1186/s12885-026-16659-y (PMC13654211; doi:10.1186/s12885-026-16659-y)
Supplement: Supplementary file 2 [file 12885_2026_16659_MOESM3_ESM.docx]

| Case | Loss | Gain/amplification |
| --- | --- | --- |
| 1 | 1p; 7p; 10; 14; 15; 18p; 19q; 22 | 4q; 5; 8; 16p |
| 2 | 1p; 2p; 6q; 9; 10q; 13; 15; 22q |  |
| 3 | 1p; 4q; 10; 14; 15; 18p; 22 | 8q; 9p |
| 4 | 3; 4; 9; 10; 11; 13; 14; 15; 19q; 22 | 6,8 |
| 5 | 2; 5q; 6; 9; 10; 14; 21; 22 | 7; 8; 13; 16; 18; 19; 20 |
| 6 | 1p; 6; 9; 14; 15; 17; 22 | 5q |

**Additional File 3**. Chromosomes with large-scale (> 5Mb) CNV in primary GISTs.
