# Supplementary material for: Establishment and genomic validation of novel patient-derived xenograft models for drug discovery in gastrointestinal stromal tumor
Source: BMC Cancer. 2026 Aug 10;26:1213. doi: 10.1186/s12885-026-16659-y (PMC13654211; doi:10.1186/s12885-026-16659-y)
Supplement: Supplementary file 3 [file 12885_2026_16659_MOESM6_ESM.docx]

| Case | Sample | CNV events^a^ | Chromosomes affected^b^ (N) | CNV fraction^c^ |
| --- | --- | --- | --- | --- |
| 1 | Primary | 23 | 12 | 35 |
|  | P0 | 17 | 12 | 35.1 |
|  | P4 | 19 | 12 | 34.9 |
|  | P8 | 26 | 13 | 35.4 |
|  | P13 | 24 | 12 | 35.2 |
| 2 | Primary | 13 | 9 | 28.5 |
|  | P0 | 10 | 10 | 26.2 |
|  | P4 | 22 | 16 | 46.7 |
| 3 | Primary | 16 | 9 | 27.5 |
|  | P0 | 11 | 9 | 26 |
|  | P2 | 16 | 9 | 25.7 |
| 4 | Primary | 13 | 12 | 41.3 |
|  | P0 | 20 | 20 | 85.4 |
|  | P2 | 22 | 19 | 76.7 |
| 5 | Primary | 18 | 15 | 56.6 |
|  | P0 | 22 | 16 | 61 |
|  | P2 | 25 | 18 | 67.7 |
| 6 | Primary | 28 | 19 | 31.7 |
|  | P0 | 14 | 10 | 35.2 |
|  | P2 | 13 | 11 | 35.1 |

**Additional File 6**. Measures of CNV prevalence across sample set

(a) Number of discrete CNV segments, (b) Chromosomes with large (>5 Mb) CNV, (c) Fraction of genome with CNV
