# Supplementary material for: Establishment and genomic validation of novel patient-derived xenograft models for drug discovery in gastrointestinal stromal tumor
Source: BMC Cancer. 2026 Aug 10;26:1213. doi: 10.1186/s12885-026-16659-y (PMC13654211; doi:10.1186/s12885-026-16659-y)
Supplement: Supplementary file 4 [file 12885_2026_16659_MOESM7_ESM.docx]

**Additional File 7**. Clustering of primary and PDX samples based on deleterious mutational signatures reveals similarity between samples belonging to the same case. Deleterious variants in cancer associated genes are labeled at DNA-notation. Data were acquired with targeted NGS using the Illumina TruSight Oncology 500 assay, filtered for deleterious variants and then clustered with the hierarchical clustering method using average Pearson correlation as distance measure.
