# Supplementary material for: Establishment and genomic validation of novel patient-derived xenograft models for drug discovery in gastrointestinal stromal tumor
Source: BMC Cancer. 2026 Aug 10;26:1213. doi: 10.1186/s12885-026-16659-y (PMC13654211; doi:10.1186/s12885-026-16659-y)
Supplement: Supplementary file 5 [file 12885_2026_16659_MOESM8_ESM.docx]

A.

B.

**Additional File 8**. Tumor growth dynamics in GIST PDX models treated with sunitinib.

(A) Model based tumor growth curves for vehicle and sunitinib treated tumors in three TKI resistant GIST PDX models. Lines represent model estimated mean tumor volumes (mm³) and shaded areas represent the standard error of the estimate. Statistical significance reflects differences in linear mixed model estimated growth rates (slopes) on the log scale. Case 2 showed a significant reduction in tumor growth rate with sunitinib compared with vehicle (slope p = 0.024). Cases 3 and 6 showed reduced growth trends under sunitinib treatment but did not reach statistical significance in slope comparisons within the study period.

(B) Individual mouse tumor growth trajectories shown as log2 transformed tumor volumes (mm³) for vehicle and sunitinib treated groups. Each line represents one mouse measured longitudinally across the study period. These plots illustrate the variability among individual tumors underlying the group level trends shown in panel A.
