# Supplementary material for: Establishment and genomic validation of novel patient-derived xenograft models for drug discovery in gastrointestinal stromal tumor
Source: BMC Cancer. 2026 Aug 10;26:1213. doi: 10.1186/s12885-026-16659-y (PMC13654211; doi:10.1186/s12885-026-16659-y)
Supplement: Supplementary file 6 [file 12885_2026_16659_MOESM9_ESM.docx]

| **Case** | **Day** | **P value** | **Label** |
| --- | --- | --- | --- |
| Case 2 | 0 | 0.5560 |  |
| Case 2 | 4 | 0.1399 |  |
| Case 2 | 7 | 0.0387 | * |
| Case 2 | 11 | 0.0099 | ** |
| Case 2 | 14 | 0.0057 | ** |
| Case 2 | 18 | 0.0045 | ** |
| Case 2 | 21 | 0.0045 | ** |
| Case 3 | 0 | 0.1506 |  |
| Case 3 | 4 | 0.0959 |  |
| Case 3 | 7 | 0.0723 |  |
| Case 3 | 11 | 0.0546 |  |
| Case 3 | 14 | 0.0474 | * |
| Case 3 | 18 | 0.0426 | * |
| Case 3 | 21 | 0.0410 | * |
| Case 6 | 0 | 0.6879 |  |
| Case 6 | 3 | 0.6203 |  |
| Case 6 | 7 | 0.5407 |  |
| Case 6 | 10 | 0.4885 |  |
| Case 6 | 14 | 0.4281 |  |
| Case 6 | 17 | 0.3891 |  |
| Case 6 | 23 | 0.3250 |  |

**Additional File 9**. Statistical comparison of tumor volumes between vehicle and sunitinib treated groups at individual time points.

P values were calculated from pairwise comparisons of treatment groups at each measurement day using the linear mixed model described in Methods, with Holm correction for multiple testing. The table lists days at which statistically significant differences were observed between groups. Asterisks denote significance levels (* p < 0.05, ** p < 0.01). No individual day-wise comparisons reached statistical significance in Case 6.
